# Notices

**Published:** 1867-06

**Authors:** 


### NOTICES.

POLITICS AND RELIGION.—The Hon. Henry Wilson, United States senator from Massachusetts, has written a book for the American Tract Society, 28 Cornhill, Boston, and 13 Bible House, New York, entitled "Testimonies of American Statesmen and Jurists to the Truths of Christianity." Sent, post-paid, for twenty cents, being an address delivered before the Young Mens' Christian Association of Natick, Dec. 23d, 1866; and we don't know that we can do better than occupy a page or two in recording the testimonies of some of the greatest men in our nation's history, in favor of the Christian religion.

Adams, John Hancock, Joseph Warren, James Otis, Elbridge Garry, Dr. Rush, &c., &c., were in favor of religion of the Bible, and the superintending providence of God in the affairs of life, and the wisdom and duty of looking up to Him for guidance and protection is given, making it a valuable little book. For sale by Messrs Broughton & Wyman, No. 13 Bible House, New York, who also have published "Lottie Wild's Pic-Nic :" 198 pp., in beautiful binding and large, clear type ; 75 cts. Contents ; Lottie's Frill, New Home, Meeting at School, Blackberry Party, Sabbath School Concert, &c., ending, "Happy Lottie, you, like Mary, have chosen that good part that shall never be taken away from you."

The medical profession throughout the United States will be glad to learn that through the enterprise of Henry C. Lea, of Philadelphia, the republication of THE HALF YEARLY ABSTRACT of the medical sciences has been resumed. It is an analytical and critical digest of the principle British and Continental medical works published in the preceding six months. It is published twice a year at $2.50, or $1.50 each ; Vol. 44. from July to December of 1866, contains 156 articles on various medical subjects, besides 63 others of short notices of reviews, books, &c., interesting to the profession. "The Abstract," with the American Journal of Medical Sciences, (itself $5 a year) with the Medical News and Library, ($1 a year) all three, sent postage free for six dollars a year. Address Henry C. Lea, Publisher, Philadelphia, Penn.

GOOD ENGLISH, or popular errors in language, by Edward S. Gould. Published by W. J. Widdleton, No. 17 Mercer st., New York. 228 pp., 12 mo., $1.50.

Mr. Gould, in this volume, has confined himself to the exposure and analysis of such philological errors as are familiar to every one, and are in common use by every one, including the best writers of England and the United States. He has omitted a great many imaginary, or possible instances of error on the ground of their being of comparatively little importance. His plan differs essentially from all the other philological works hitherto published, both in the limitation of subjects and in the manner of treating them. The reader will be surprised to find how many errors have crept into the language and received the sanction of the usage of good writers; and also he will be surprised to find how entirely incorrect and corrupt are many words and expressions that everybody seems to suppose are unquestionably good English.

VACCINATION.—J. P. Loines, House Physician, Eastern Dispensary, 57 Essex St., New York, and Bullock & Henshaw, corner of Arch and 6th Sts., Philadelphia, Pa., always keep on hand the purest, best and freshest vaccine matter. This notice is given gratuitously for the benefit of our readers. One dollar will secure enough matter to vaccinate a dozen persons.

CHEAP ICE PITCHER.—In many parts of the country ice has to be brought a great distance for the sick. It is preserved a very long time by putting it in a jar or pitcher, then take a length of thick brown paper, or several thicknesses of newspaper, sufficient to reach round the vessel; it should be broader than the vessel is high; to the edge of the paper attach with a thread and needle a piece of cotton batting three inches thick; close one end of this long, round box with a similar material, then set the vessel of ice in it, and fold over the extra length so as to exclude the air; or cover it with a pillow.

MARK TWAIN, who spent some months in the Sandwich Islands, delivered a lecture on the character of the country and people in the large hall of the Cooper Institute lately, to a very large assembly. It seemed to us that there was not a vacant seat. It was one of the most interesting and irresistably amusing lectures we have ever heard on that or a similar subject. The warm and outspoken testimony given of the fidelity and success of the American Missionaries, received rapturous applause. We advise every reader to be at some pains to hear this lecture, should it be delivered in the Provinces! One sad statement was made. Eighty years ago, they numbered eight hundred thousand souls, now less than sixty thousand, the result of the introduction of liquor, small-pox, and the diseases of licentiousness introduced by the sailors of foreign vessels calling at the Islands. Another most interesting fact was elicited, that it was the finest sugar country on the globe. Six years ago the planting of sugar cane was made a business,and three million pounds of sugar were produced; last year, if we heard rightly, it was thirty millions ; twelve or fifteen hundred pounds to the acre is considered a good crop in Louisiana, but the soil of these islands will yield as many thousand pounds. It is the finest climate in the world ; the thermoneter does not vary a dozen degrees in a year, and ranges from seventy to eighty. By all means, reader, hear this most remarkable lecturer, he will give you laugh enough to keep you in vigorous health for a long time to come.

" DOMES OF THE GREAT YOSEMITE."—This is Bierstadt's new picture, on exhibition at 51 West Tenth St., New York, for the benefit of the New York Ladies' Southern Relief Association. There is a beauty and grandeur about this painting which at once stamp the artist as possessing a master hand ; there are features in it which admit of study by the hour, and we go away wondering at the greatness of nature and the pencil, which can with so much fidelity, transfer to canvas the likeness of the reality.

The American Tract Society have issued " George Wayland, the Little Medicine Carrier," from the Religious Tract Society of London ; 103 pp. ; a beautiful narration of childish suffering and patience and piety. Also, the Cinnamon Isle Boy, by Mrs. E. C. Hutchings ; 169 pp. It is the history of Charles, son of Rev. Miron and Harriet L. Winslow, born in Ceylon, 1821, died in New York, 1832, buried in the graveyard at New Haven, Conn., having slept in Jesus as all do who " early seek his face."

A beautiful and most instructive and useful book of 359 pp. is THE HOPES OF HOPE CASTLE, or the Times of John Knox and Queen Mary Stuart, by Mrs. S. T. Martin, author of the Women of the Bible, Allan Cameron, &c. ; being a journal kept by Kathel Hope for her friend, Ellen Maxwell ; telling about John Knox and Mary Queen of the Scots and Rizzio's murder, and much more besides of what took place in those eventful days. It is a book of very great interest.

" PAUL VENNER," or the Forge and Pulpit, based on facts, is another volume of the same society, at 150 Nassau St., New

York, 371 pp. It is a story of facts, and is one of those illustrations of life well calculated to inculcate useful, practical lessons, and to show how to live successfully and to die respected, beloved and lamented.

Messrs. Broughton & Twyman of 13 Bible House, New York, have sent us some of the recent issues of the American Tract Society of 28 Cornhill, Boston. Jonah, the Prophet; Lessons on his Life, by Professor Gaussen, being addresses delivered to a Sunday school at Geneva ; pp. 167. Also a very instructive volume of 208 pages, entitled " Glimpses of West Africa," with sketches of missionary labor, by Rev. Samuel J. Whiton, written during odd moments of missionary life, relating mostly to the country and tribes near the Mendi Mission, seven degrees north latitude ; it is an authentic narration and deserves to be made a standard publication of historical facts. Also, the "Honorable Club," and other tales by Lynde Palmer, the author of Helps Over Hard Places, 270 pp. ; also, " Following the Leader," 247 pp., showing to children how beautiful a thing it is to obey the injunction of our Lord implied in the utterance, " If any man will serve me, let him follow me ;" also, " A Sister's Story," 298 pp., to be read alike by daughters and sons, by girls and boys, closing with the suggestive statement, " when you have done all that mortal skill and love can do, God will take up your task and reward you with its sure and glorious completion."

The Bankrupt Law of the United States 1867 ; with Notes and a Collection of American and English Decisions upon the Principles and Practice of the Law of Bankruptcy, Adapted to the Use of the Lawyer and Merchant. By Edwin James, of the New York Bar, and one of the Framers of the Amendment Act. Harper & Brothers.

This octavio, we presume, will become the standard volume on the recent Bankrupt Law. The text is printed in very large type, and the commentary follows each separate section, making the whole matter intelligible to the ordinary reader.

Harper's Weekly, $4 a year ; Harper's New Monthly Magazine, $4.

